# Rhino defines H3K9me3-marked piRNA clusters

**DOI:** 10.18632/oncotarget.5178

**Published:** 2015-08-13

**Authors:** Bowen Yu, Ying Huang

**Affiliations:** National Center for Protein Science Shanghai, State Key Laboratory of Molecular Biology, Shanghai Key Laboratory of Molecular Andrology, Institute of Biochemistry and Cell Biology, Shanghai Institutes for Biological Sciences, Chinese Academy of Sciences, Shanghai, China

**Keywords:** piRNA cluster, Rhino, chromodomain

piRNAs (PIWI-interacting RNAs), which are generally 24-32 nucleotides in length, are defined by their specific association to the PIWI subfamily of AGO/PIWI family proteins. piRNAs are mainly made in animal gonads, and are essential to silence transposons during germ line development [1]. In *Drosophila,* the genomic regions that transcribe piRNA precursors are named as piRNA clusters. The primary piRNAs are transcribed from piRNA clusters. Most of the piRNA clusters are located in pericentromeric and subtelomeric heterochromatin regions coated with H3K9me3 mark. There are two types of piRNA clusters in the *Drosophila* ovaries: uni-strand and dual-strand, which are mainly active in somatic follicle cells and germ cells, respectively. The mechanisms defining the uni-strand or dual-strand piRNA clusters are different. Recent studies have shown that Rhino, a germline-specific protein required for primary piRNA production in germline cells, is anchored to H3K9me3-marked chromatin and specifically binds to dual-strand clusters [2, 3].

Rhino belongs to the heterochromatin protein 1 (HPl) family, also called HPlD. Like other proteins in the HPl family, Rhino contains two conserved domains: N-terminal chromodomain (CD) and C-terminal chromoshadow domain (CSD), and a hinged region in between. The chromodomain is in charge of recognizing the H3K9me3 marker, and the chromoshadow domain is involved in interaction with other proteins. The hinged region in Rhino is much longer than in other members of this family. Here, the molecular features that distinguish Rhino from other HPl proteins in *Drosophila* and allow it to specifically recognize dual-strand piRNA clusters are found.

We and Le Thomas *et al.* have recently reported the high resolution structure of Rhino chromodomain (Rhino CD) in complex with H3K9me3 [4, 5]. Surprisingly, the Rhino-CD forms a dimer both in crystal and in solution, which is unique among all known chromodomains. Each monomer has an aromatic cage binding H3K9me3, a common feature shared by the chromodomains from other HPl family proteins. But the dimeric Rhino-CD allows the binding of two H3K9me3 peptides in anti-parallel manner. Both aromatic cages and dimerization of Rhino-CD were found to be important to its function both in *vitro* and in vivo. The Rhino-CD mutants lost binding ofH3Kc9me3-labeled polynucleosomes, a mimic of the H3K9me3-polynucleosomes. The corresponding mutation leads to transposon activation and fly sterility [4].

Considering that Rhino specifically locates to dual-strand clusters rather than uni-strand clusters, a special chromatin structures is suggested. Yamanaka *et al.* have recently proposed a possible link between chromatin boundaries and piRNA clusters [6]. H2A.Z and H3.3 are two conserved histone variants and usually located at gene promoters, enhancers and chromatin boundaires as an indicator of open chromatin conformation. Interesting, both H2A.Z and H3.3 were the genome-wide RNAi screen hits that can recover the transposon silencing in Drosophila [6]. speculated that both histone variants are involved in piRNA cluster formation in the open chromatin boundary regions [6]. However, little is known about the chromatin structures, much less the boundary chromatin structures. Based on the recently published 3D cryo-EM structure of 30 nm chromatin fiber [7], a model of how Rhino-CD dimer may recognize the piRNA cluster by two histone tails from the nucleosomes on both strands has been proposed. The binding of Rhino may facilitate the packing of chromatin fiber and stabilization of the chromatin structure.

An additional finding of the current study was the DNA binding affinity of Rhino-CD. In the binding model proposed here, the Rhino is probably located in the groove ofthe double helix ofthe 30-nm chromatin fiber. Sequence alignment showed the C-terminal helix to be longer and positive charged, which renders it quite different from other chromodomain proteins. The C-terminal helixes of the Rhino-CD dimer form a positively charged clamp shaped conformation, which may facilitate the association of the linker dsDNA connecting neighboring nucleosom es and stabilization of Rhino binding. The binding of Rhino to both dsDNA and H3K9me3 makes it more feasible to recognize piRNA clusters than other the canonical HPl proteins.

After almost ten years of research, the importance of piRNAs for germline cell development has become abundantly clear. However, the precise molecular details of how piRNAs are produced and how the pathway represses mobile elements remain poorly understood. Our work would shed light on the mechanism of the recruitment of Rhino to the piRNA cluster regions. However, the structural features of chromatin in piRNA cluster regions recognized by Rhino that facilitate the transcription of dual-strand clusters still need further study.
